# The Importance of Follow-Up and Evaluation of Intraoperative Findings to Determine Surgical Indications for Retractile Testis

**DOI:** 10.1155/2023/8764631

**Published:** 2023-09-08

**Authors:** Kazuro Kikkawa, Yuko Ueda, Shimpei Yamashita, Yasuo Kohjimoto, Isao Hara

**Affiliations:** Department of Urology, Wakayama Medical University, 811-1 Kimiidera, Wakayama 641-0012, Japan

## Abstract

**Objectives:**

Ascending testis or acquired undescended testis develops in approximately 30% of cases of retractile testis, and orchiopexy is recommended for these cases. This study aimed at assessing the intraoperative anatomical findings of ascending testis and acquired undescended testis in search of better management for retractile testis.

**Methods:**

We retrospectively collected data of patients with confirmed diagnosis of retractile testis between February 2012 and November 2021. Orchiopexy was performed for cases with ascending testis and for patients with increasing difference of right and left testicular volume. The site of gubernaculum attachment and patent processus vaginalis were evaluated during surgery.

**Results:**

A total of 119 testes in 71 patients with retractile testis were included in this study. Sixteen retractile testes in 12 patients (17%) underwent orchiopexy. The weight at birth was significantly higher, and bilateral retractile testes were significantly more common in the follow-up group than in the surgical intervention group. In the surgical intervention group, the abnormal site of gubernaculum attachment was found in 12 out of 16 testes (75%), and patent PV was found in nine out of sixteen testes (56%). Sites of gubernaculum attachment in testes with patent PV were significantly higher than in sites with closed processus vaginalis, and all testes with patent processus vaginalis had abnormal site of gubernaculum attachment.

**Conclusion:**

Patients with ascending testis and acquired undescended testis have clinical features and intraoperative abnormal findings similar to a cryptorchidism. Therefore, our surgical indication for retractile testis is considered appropriate.

## 1. Introduction

Retractile testis (RT) is defined as the testis located in the upper scrotum or groin that can descend into the lower scrotum spontaneously or with manipulation and remain there for a period of time [1]. RT is traditionally considered to be a normal variant of testis because RT usually descends into the scrotum during adolescence, but observation of testicular position is recommended [2, 3]. However, ascending testis (AT) or acquired undescended testis (UDT) develops in about 30% of cases of RT [4]. RT can be difficult to distinguish from acquired UDT. The development mechanism of AT and acquired UDT in RT may be associated with anatomical anomalies, including the site of gubernaculum attachment and patent processus vaginalis (PV) [5, 6]. RT should therefore be carefully observed, and orchiopexy is recommended for AT and acquired UDT. However, it is difficult for urologists to decide whether or not orchiopexy is necessary.

We investigated the natural course and outcome of RT, including spontaneous resolution and surgical intervention, at a single institution in Japan. The aim was to assess the anatomical anomalies and validity of surgical indication associated with RT with the intention of elucidating better management for RT.

## 2. Materials and Methods

We retrospectively collected data of patients who had confirmed diagnosis of RT at Wakayama Medical University, Japan, between February 2012 and November 2021. All patients underwent physical examination and ultrasonography (US) by experienced urologists. RT was defined as the testis located in the upper scrotum or groin that could manually descend into the lower scrotum without tension and remain there for a period of time. These patients with RT were followed every six months or one year by the same urologist. Surgical indications were AT or increasing difference in testicular volume between right and left during the duration of follow-up. AT was defined as testis located in the upper scrotum or groin that could not descend into the lower scrotum and immediately return to its original position. Acquired UDT was defined as the testis that could descend to the bottom of the scrotum manually with smaller testicular volume compared with the contralateral testis and/or testicular volume decrease during the course of follow-up. The testicular volume was measured by ultrasound. The standard orchidopexy was performed under general anesthesia for all patients eligible for surgical intervention. The operation involved a groin skin incision and a scrotal incision with fixation of the testis in tunica dartos in all cases. There was no case that performed the testicular biopsy. The site of gubernaculum attachment was evaluated by pulling the testis under gentle traction during surgery. Furthermore, the presence of a patent PV was identified by dissection of the aponeurosis of external oblique muscle up to the inner inguinal ring. Resolution of RT was defined as the testis that had descended into the lower scrotum spontaneously and remained in the scrotum, following induction of cremasteric reflex.

Patients were divided into two groups based on management as follow-up or surgical intervention. Patients in the follow-up group were further divided into two groups based on the outcome as follows: continuation of follow-up or spontaneous resolution. The clinical data were retrospectively reviewed, including patient characteristics, duration of follow-up, physical findings, and intraoperative findings. The duration of follow-up in the surgical intervention group was regarded as the period from the first diagnosis of RT to surgery. Data of the two groups were compared to assess the difference in the outcome of RT. This study was conducted with the approval from the Wakayama Medical University Institutional Review Board (approval number: 3723) and in accordance with the Declaration of Helsinki.

Statistical analysis was performed using JMP Pro 14 (SAS Institute Inc., Cary, NC, USA). The Wilcoxon signed rank test was used to compare continuous variables between the two groups, and the chi-square test and Fisher's exact test were used for categorical variables. *P* values <0.05 were considered significant.

## 3. Results

A total of 119 testes in 71 patients were diagnosed with RT and included in this study. Included in the follow-up group were 103 retractile testes (RTs) in 59 patients (83%), and surgical intervention was performed in 16 RTs in 12 patients (17%). Patient characteristics of the two groups are shown in Table 1. The mean (interquartile range) ages at the time of diagnosis were 1 (0–3) years in both groups (*P*=0.706). Weight at birth in the surgical intervention group was significantly lower than that in the follow-up group. The bilateral RTs of the follow-up group were significantly more common than in the surgical intervention group. The mean (interquartile range) duration of follow-up was 28 (12–46) months in the follow-up group and 25 (6–38) months in the surgical intervention group (*P*=0.276).

In the follow-up group, 81 testes in 45 patients continued to have RT at the most recent examination and continued to be followed up (Table 2). There was spontaneous resolution in 22 RTs in 14 patients. The weight at the time of birth in the patients with spontaneous resolution was lower than that of the patients with continuation of follow-up, but there was no significant difference between the two groups. The location of RT at diagnosis in the patients with continuation of follow-up was slightly higher than that in the patients with spontaneous resolution, but there was no significant difference between the two groups. Duration of follow-up in the patients with spontaneous resolution was significantly longer than in the patients with continuation of follow-up.

In the surgical intervention group, eight testes in seven out of twelve patients were diagnosed with AT and eight testes in five patients had an increasing difference between the right and left testicular volume without AT as acquired UDT (Table 3). The abnormal site of gubernaculum attachment was found in 12 out of 16 testes (75%), and patent PV was found in nine out of sixteen testes (56%). AT had no significant association with the site of gubernaculum attachment or PV. Patent PV in the testis of unilateral RT was significantly more common in the testis of bilateral RT (Table 4). The sites of gubernaculum attachment in the testes with patent PV were significantly higher than in the testes with closed PV, and all the testes with patent PV had an abnormal site of gubernaculum attachment. All AT had anatomical abnormalities, but three of sixteen testes (19%) had closed PV and normal site of gubernaculum attachment with acquired UDT.

## 4. Discussion

Some causes of RT have been reported, including an overactive cremasteric reflex [7]. Although observation of the testis is recommended as the management of RT, about 30% of RT cases become AT or acquired UDT [4]. Defining RT as a “normal variant” may therefore be controversial. In this study, 17% of RT patients were diagnosed with AT or acquired UDT and required surgical intervention. A lower incidence of AT and acquired UDT in this study than that in the previous reports may be due to the shorter observation period of RT in the follow-up group without spontaneous resolution. In this study, the birthweight of patients that received surgical intervention was significantly lower than that of patients that only received follow-up. Cryptorchidism is more common among preterm boys and boys whose weight at birth is under 2500 g [8]. These findings and those of the previous reports suggest that pathophysiology of RT with the development of AT or acquired UDT may be similar to cryptorchidism. In the current study, significantly more patients with bilateral RTs received only follow-up than those who underwent surgical intervention. Bilateral RT was previously reported as a predictive factor for spontaneous resolution [9]. It was suggested that bilateral RT may occur as a result of bilateral hyperactive cremasteric reflex and eventually move to the bottom of the scrotum. Cremasteric reflexes differ according to age, the highest incidence being in boys aged between five and eight years old [10]. Diagnosis of RT may therefore be more difficult at these ages, and careful follow-up is required.

AT is defined as a previously descended testis that cannot be manipulated into the scrotum [11]. In addition, if RT can be manipulated down to the scrotum with smaller testicular volume compared with the contralateral testis, this condition should also be considered as acquired UDT [4, 12]. AT and acquired UDT resulting from RT are thought to be related to anatomical abnormalities as cryptorchidism [5, 6, 13]. The presence of patent PV may cause AT and acquired UDT because patent PV itself can stunt elongation of the cord structures and placement of the testis in the scrotum [5]. In addition, the absorption of PV into parietal peritoneum may lead to traction on the cord and there may be development of AT or acquired UDT [5, 13]. The abnormal site of gubernaculum attachment may also be a cause of AT and acquired UDT [6]. A proximal site of gubernaculum attachment can be a factor in AT and acquired UDT, with the testis being pulled up into the groin during growth of a boy with RT. Rabinowitz and Hulbert described that an abnormal site of gubernaculum attachment was a more consistent finding than patent PV in boys with AT from RT [14]. In the current study, the abnormal site of gubernaculum was actually more common than patent PV in AT and acquired UDT, requiring surgical intervention. This finding and previous reports support the hypothesis that an abnormally inserting gubernaculum could pull the testis from a scrotal position up into the groin during the course of growth [6].

One hypothesis suggests that a fibrous remnant of the normally closed PV remains tethered to the testis and peritoneum [5, 13]. The cord of the RT with this fibrous remnant fails to elongate, and the testis moves out of the scrotum. In the current study, neither patent PV nor abnormal site of gubernaculum attachment were recognized in the three testes with surgical intervention for increasing differences in testicular volume. These testes might have a fibrous remnant of the closed PV. However, a fibrous remnant is often based on the surgeon's impression, and the findings of RTs with surgical intervention in this study were not recorded and unclear.

This study has several limitations. Patient data were retrospectively collected from a single institution, and the sample size was small. Another limitation was the risk of bias in diagnosis of AT or acquired UDT and differentiating them from cryptorchidism. However, we have confidence in the diagnosis of AT and acquired UDT in this study because patients were examined by two urologists with a strict and consistent definition of RT. In addition, 45 out of 71 patients (63%) in this study still had RT without spontaneous resolution at the most recent visit. Further observation is therefore necessary to more accurately evaluate the distinctive features of RT to identify the predictive factors for spontaneous resolution, AT or acquired UDT. Furthermore, intraoperative findings including absorption into the parietal peritoneum and fibrous remnant of the PV should be recorded in detail to gain a deeper understanding of AT and acquired UDT from RT.

In conclusion, although RTs are considered a variant of normal testes, surgical intervention is required in some cases with AT or acquired UDT. Patients with AT and acquired UDT have clinical features similar to a cryptorchidism, including weight at birth and laterality. In addition, intraoperative abnormal findings of AT and acquired UDT, including patent PV and abnormal site of the gubernaculum, seem to share similarity with cryptorchidism. Therefore, surgical indication for RT, including nondescending testis by manual and increasing differences in testicular volume, is considered appropriate in this study. To avoid misdiagnosis, screening failures, and unnecessary surgery, it is important to acquire the follow-up and skills for examinations to investigate AT and acquired UDT with a significant risk of anatomical anomalies. A better understanding of RT obtained by further investigation may lead to the development of better management.

## Figures and Tables

**Table 1:** Characteristics of patients and testes.

	Follow-up		Surgical intervention		*P* value
	*n* = 59		*n* = 12		
	(103 testes)		(16 testes)		
Age at diagnosis, median years (IQR)	1	(0–3)	1	(0–3)	0.706
Gestational age, median weeks (IQR)	38	(36–39)	38	(30–39)	0.313
Weight at birth, median grams (IQR)	3073	(2574–3358)	2325	(1075–2936)	0.025
Laterality					0.014
Unilateral, *n* (%)	15	(25)	8	(67)	
Bilateral, *n* (%)	44	(75)	4	(33)	
Side (119 testes)					0.914
Right, *n* (%)	50	(49)	8	(50)	
Left, *n* (%)	53	(51)	8	(50)	
Location (119 testes)					0.790
Upper scrotum, *n* (%)	51	(49)	7	(44)	
Groin, *n* (%)	52	(51)	9	(56)	
Duration of follow-up, median month (IQR)	37	(24–58)	25	(6–38)	0.138

IQR = interquartile range.

**Table 2:** Characteristics of patients and testes in the follow-up group.

	Continuation of follow-up		Spontaneous resolution		*P* value
	*n* = 45		*n* = 14		
	(81 testes)		(22 testes)		
Age at diagnosis, median years (IQR)	1	(0–3)	2	(1–3)	0.544
Gestational age, median weeks (IQR)	38	(36–39)	38	(24–40)	0.731
Weight at birth, median grams (IQR)	3109	(2790–3365)	2480	(644–3192)	0.057
Laterality					0.156
Unilateral, *n* (%)	9	(20)	6	(43)	
Bilateral, *n* (%)	36	(80)	8	(57)	
Side (103 testes)					0.878
Right, *n* (%)	39	(48)	11	(50)	
Left, *n* (%)	42	(52)	11	(50)	
Location at diagnosis (103 testes)					0.057
Upper scrotum, *n* (%)	36	(44)	15	(68)	
Groin, *n* (%)	45	(56)	7	(32)	
Duration of follow-up, median month (IQR)	30	(23–50)	56	(29–98)	0.015

IQR = interquartile range.

**Table 3:** Intraoperative findings of the surgical intervention group.

Age at surgery, median years (IQR)	4	(3–5)

*Surgical indication*		
Ascending testis, *n* (%)	7	(58)
Differences in testicular volume, *n* (%)	5	(42)

*Site of gubernaculum attachment (16 testes)*		
Normal, *n* (%)	4	(25)
Upper scrotum, *n* (%)	3	(19)
Groin, *n* (%)	9	(56)

*Processus vaginalis (16 testes)*		
Closed, *n* (%)	7	(44)
Patent, *n* (%)	9	(56)

IQR = interquartile range.

**Table 4:** Comparison of testicular characteristics depending on the state of processus vaginalis.

	Closed		Patent		*P* value
	*n* = 7		*n* = 9		
*Laterality*					0.041
Unilateral, *n* (%)	1	(14)	7	(78)	
Bilateral, *n* (%)	6	(86)	2	(22)	

*Location at diagnosis*					0.615
Upper scrotum, *n* (%)	4	(57)	3	(33)	
Groin, *n* (%)	3	(43)	6	(67)	

*Surgical indication*					0.315
Ascending testis, *n* (%)	2	(29)	6	(67)	
Difference in testicular volume, *n* (%)	5	(71)	3	(33)	

*Site of gubernaculum attachment*					0.020
Normal, *n* (%)	4	(57)	0	(0)	
Upper scrotum, *n* (%)	0	(0)	3	(33)	
Groin, *n* (%)	3	(43)	6	(67)	

## Data Availability

The retrospective data used to support the findings of this study are included within the article.

## Abbreviations

RT: Retractile testis
AT: Ascending testis
UDT: Undescended testis
PV: Processus vaginalis
US: Ultrasonography
RTs: Retractile testes.
